# Controlling epidemic spread by social distancing: Do it well or not at all

**DOI:** 10.1186/1471-2458-12-679

**Published:** 2012-08-20

**Authors:** Savi Maharaj, Adam Kleczkowski

**Affiliations:** 1 Computing Science and Mathematics, University of Stirling, Stirling FK9 4LA, United Kingdom

## Abstract

**Background:**

Existing epidemiological models have largely tended to neglect the impact of individual behaviour on the dynamics of diseases. However, awareness of the presence of illness can cause people to change their behaviour by, for example, staying at home and avoiding social contacts. Such changes can be used to control epidemics but they exact an economic cost. Our aim is to study the costs and benefits of using individual-based social distancing undertaken by healthy individuals as a form of control.

**Methods:**

Our model is a standard SIR model superimposed on a spatial network, without and with addition of small-world interactions. Disease spread is controlled by allowing susceptible individuals to temporarily reduce their social contacts in response to the presence of infection within their local neighbourhood. We ascribe an economic cost to the loss of social contacts, and weigh this against the economic benefit gained by reducing the impact of the epidemic. We study the sensitivity of the results to two key parameters, the individuals’ attitude to risk and the size of the awareness neighbourhood.

**Results:**

Depending on the characteristics of the epidemic and on the relative economic importance of making contacts versus avoiding infection, the optimal control is one of two extremes: either to adopt a highly cautious control, thereby suppressing the epidemic quickly by drastically reducing contacts as soon as disease is detected; or else to forego control and allow the epidemic to run its course. The worst outcome arises when control is attempted, but not cautiously enough to cause the epidemic to be suppressed. The next main result comes from comparing the size of the neighbourhood of which individuals are aware to that of the neighbourhood within which transmission can occur. The control works best when these sizes match and is particularly ineffective when the awareness neighbourhood is smaller than the infection neighbourhood. The results are robust with respect to inclusion of long-range, small-world links which destroy the spatial structure, regardless of whether individuals can or cannot control them. However, addition of many non-local links eventually makes control ineffective.

**Conclusions:**

These results have implications for the design of control strategies using social distancing: a control that is too weak or based upon inaccurate knowledge, may give a worse outcome than doing nothing.

## Background

Network models have been successfully used to describe the spread of many infectious diseases, ranging from human [1-8] through animal [6,9-11] to plant diseases [12]. In these models, individuals are represented as nodes and potential contacts between individuals as edges of the underlying network. Much work has been devoted to studying how networks are assembled [13-15] and to analysing the effect of network topology on disease spread and potential control strategies [16]. However, the structure of interactions between individuals is most commonly assumed to be fixed and so represents an ‘average’ behavioural pattern. Thus, conventional epidemiological models either treat human behaviour as external to the disease system or even ignore it completely [17,18].

The structure of real-life networks is, however, far from static and often responds to epidemic spread at both individual and at population level. Thus, for example, governments often introduce control measures aimed at disrupting disease transmission either locally or on long-range links [19-21]. Targeted social distancing may be promoted by governments and public health bodies as a strategy for the control of epidemics, for example in the form of school or workplace closures. The effectiveness of such measures is typically assessed by macroeconomic cost-benefit analysis, often based upon large-scale Computational General Equilibrium models [5,22-28].

Social distancing also arises spontaneously as individuals respond to news about disease spread, obtained from media reports, public announcements, rumours or individual experience [29]. Recognising the importance of behavioural responses, epidemiological modellers have begun to consider transmission via adaptive networks [15,30,31], whereby the structure of the network is modified depending on the state of individuals. Thus, changes in contact network structure can arise as a result of human responses to disease, such as a reduction in social contacts. Such models have shown that social distancing can be effective at reducing the attack rate of an epidemic [28,32] and that it is a plausible explanation for certain phenomena arising in real epidemics, such as multiple outbreaks or waves of infection [32,33].

Existing studies have, however, tended to neglect the associated cost to both society and individuals resulting from the actions of individuals leading to severing social links. Social contacts are necessary for economic activity: employees must go to work, students must go to school, and traders and customers must interact in order for an economy to function. Social contacts are also important to individuals for non-monetary reasons, such as interactions with family and friends. Awareness of an epidemic therefore presents each individual with a private choice between investing in social contacts and risking infection, or reducing the contacts and losing the social or economic benefits [34]. This can have a severe impact on both individual and societal well-being as it has been noted that for example ‘(..) fear of exposure can result in significant worker absenteeism’ [35].

In this paper we study the economic impact of social distancing as a disease control strategy undertaken by healthy individuals in response to either local information about the number of cases in their immediate neighbourhood or global information communicated by mass media. We take into account two factors: the economic cost of individuals falling ill, and the economic cost of reduction in social contacts. For simplicity we do not distinguish between contacts for different purposes, treating all contacts as if they have the same economic worth. Our model is based upon a standard SIR model superimposed upon a simple spatial network. Computer simulations are used to study the effect of allowing susceptible individuals to reduce their contacts when their surrounding neighbourhood contains infected individuals. One important parameter we use is the *risk attitude*, which models how strongly individuals respond to the awareness of a threat. Another important parameter is the *awareness neighbourhood*, which represents the size of the area that individuals are knowledgeable about. Our main result is that, in order for social distancing to be a cost-effective strategy, it must be applied with a highly cautious attitude to risk. We also see that the size of the awareness neighbourhood matters: for best results, this should be the same as the size of the neighbourhood within which disease transmission can occur. In particular, the control is most effective if the response is based on local knowledge – the result of mass media-induced panic is to reduce the effectiveness of the spontaneous control.

In this paper we consider two network models. The first one is a regular network which has the advantage of simplicity but is only a crude representation of real social networks which often display a mixture of short-range and long-range links. We therefore also consider a small-world model that is more realistic in this respect [13,36,37], with long-range links representing occasional travel of individuals. The main result of our paper is robust to the addition of even a relatively high number of long-range links, although if the network becomes very random, the optimal response is to refrain from any action. In this case, the highly cautious risk attitude leads to a limited but long epidemic causing large losses in contact numbers as individuals repeatedly respond to the threat.

## Methods

The model of infection is based upon a standard SIR model superimposed upon a simple spatial network with neighbourhoods defined by Euclidean distance, without and with addition of long-range links. Each individual has an infection neighbourhood whose size, and hence the number of contacts made by that individual, depends locally on the infection pressure and therefore changes throughout the epidemic. The model includes an economic cost-benefit analysis which evaluates the overall benefits to the global population arising from local individual responses to infection levels.

### Spatial model

**Figure 1:**
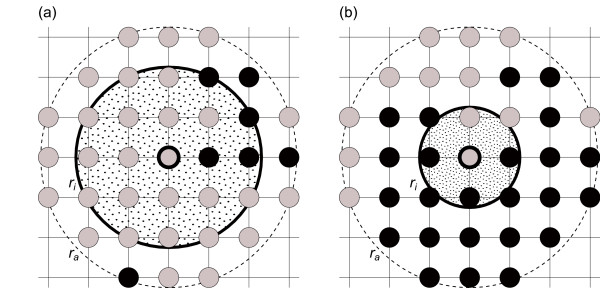
**Structure of the awareness and infection neighbourhoods for the case with a small infection pressure (a) and large infection pressure (b).** Susceptible individuals are shown as grey circles and infected individuals as black circles. For clarity no recovered individuals are shown in this example and only individuals in the awareness neighbourhood (large dotted circle) are represented. The infection neighbourhood (thick-lined circle) varies in response to increased infection pressure (cf. (**b**) with (**a**)).

For simplicity, we assume that individuals are located at nodes of a square lattice representing their geographical distribution. We assume no-contact boundary conditions, but we have found that boundary wrapping has little effect on the results. Each node of the lattice holds a single individual, which may be either *susceptible*, *infected*, or *removed/recovered*. On the lattice we define a neighbourhood of radius *r* centered at a node (*i*_0_,*j*_0_), determined by Euclidean distance, $\left\{(i,j)\;\mathrm{s.t.}\;\sqrt{{\left(i-i_{0}\right)}^{2}+{\left(j-j_{0}\right)}^{2}}\leq r\right\}$. For each individual two types of neighbourhoods are considered, describing two network topologies, see Figure 1. The *infection neighbourhood* is characterised by radius *r*_*i*_ and describes spread of the infection, whereas the *awareness neighbourhood* defines the area of influence on individual decisions, and is determined by the radius *r*_*a*_. Both radii can range from 0 (no neighbours) to infinity (whole population), although in practice infinity is represented by a finite value big enough to encompass the whole population. The awareness neighbourhood is uniform for all individuals and fixed for the duration of each epidemic, whereas the infection neighbourhood varies locally depending on the infection pressure (for details see below). The number of individuals in each neighbourhood is proportional to the radius squared and we denote by *N*_*a*_ the number of nodes in each awareness neighbourhood and by *N*_*i*_the number of nodes in each infection neighbourhood. Initially all individuals are assigned the same baseline infection neighbourhood with radius $r_{i}^{\left(0\right)}$ leading to the number of individuals $N_{i}^{\left(0\right)}$ in each infection neighbourhood.

**Figure 2:**
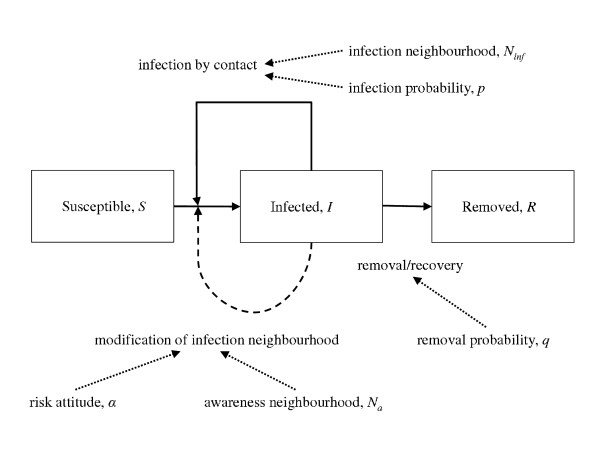
**Block diagram illustrating transitions in the model considered in the paper.** Solid lines represent transitions performed at each time step. Dashed line illustrates change in behaviour influenced by infection pressure. Dotted lines identify parameters affecting each transition.

The epidemiological model is based upon an SIR (Susceptible-Infected-Recovered) model [38] (see Figure 2). All individuals are initially susceptible (**S**) and the epidemic is initiated by the introduction of few infected individuals (**I**) at random locations in the network. Each susceptible individual is affected by all infected individuals within its infection neighbourhood independently of their distance. Each contact between an infected and a susceptible individual may result in the susceptible becoming infected, with probability *p* per contact (and per time step). At each time step, each infected individual may become recovered, with probability *q*. Recovered individuals cannot be reinfected. The epidemic ends when all infected individuals recover. The simulation proceeds through discrete time steps (so that each probability can be interpreted as a hazard) up to a maximum number of steps, chosen to be sufficiently large to allow all epidemics to run to completion. Although our model is generic and so does not depend on a choice of a particular time step, we envisage our model to apply to relatively fast-spreading epidemics. Thus, for simplicity we assume the time step equal to one day with most epidemics finished in less than 120 days.

Social distancing is introduced by allowing susceptible individuals to temporarily reduce the number of contacts they make in response to the presence of nearby infection. Thus, each susceptible individual can detect the current infection pressure amongst its neighbours within the awareness radius, *r*_*a*_. The local infection pressure, *Θ*, is the ratio of the number of infected individuals in radius *r*_*a*_to the total number of neighbours within that radius. For any given susceptible individual, *Θ*may take values ranging from 0, meaning that there are no infected neighbours in radius *r*_*a*_, to 1, meaning that all neighbours within radius *r*_*a*_are infected.

**Figure 3:**
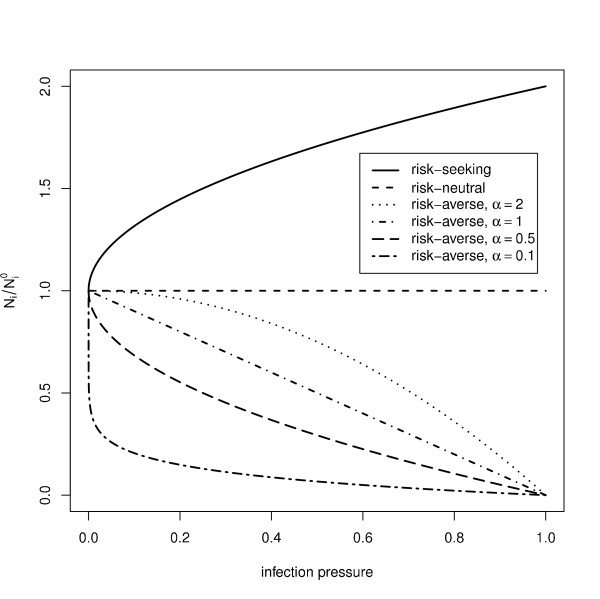
**The role of risk attitude in modifing the number of contacts in response to the local infection pressure.** The curves illustrate (from top to bottom) risk-seeking, risk-neutral, and increasingly more risk-averse.

How strongly susceptible individuals react to the current infection pressure depends upon another control parameter, *α* (risk attitude), which represents the degree of caution used in applying the control. Intuitively, when a population is more cautious, or more risk-averse, susceptible individuals will react more strongly by reducing their contacts more drastically in response to a given infection pressure. Thus, if a susceptible individual detects an infection pressure *Θ* at the current time step, then the number of contacts that susceptible will make during the next time step is adjusted by choosing a new contact radius $r_{i}^{\prime }$ and the corresponding new number of individuals within its infection neighbourhood

(1) $$N_{i}=N_{i}^{\left(0\right)}\left(1-\Theta^{\alpha }\right)\;,$$

see Figure 3. This formula ensures that if no infection is present within a susceptible’s awareness radius (*Θ*=0), it will resume full contact by reverting to the initial, maximum contact radius $r_{i}^{\left(0\right)}$. Increased local infection pressure causes the infection radius and hence the number of contacts to decrease.

Lower values of *α*represent more cautious (more risk-averse) attitudes to risk, and result in a greater reduction of *r*_*i*_ for a given *Θ*. For completeness, Figure 3 also illustrates ‘risk-neutral’ and ‘risk-seeking’ responses, represented by the upper two lines. The risk-neutral case is one in which susceptibles do not modify their behaviour, and is equivalent to no control being used: here $r_{i}^{\prime }=r_{i}^{\left(0\right)}$, regardless of the infection pressure. The risk-seeking case represents a response in which susceptibles seek to increase their contacts as the infection pressure increases, modelled by the formula $N_{i}=N_{i}^{\left(0\right)}\left(1+\Theta^{\alpha }\right)$. Risk-seeking response was not considered in this study, though we note that such seemingly perverse behaviour was observed [39] in a virtual ‘epidemic’ in an online computer game.

For contact to occur between two individuals, each one must be within the contact neighbourhood of the other. At each time step, any two individuals *a* and *b* will make contact, provided that each is within the other’s contact radius, *i.e.*, if *d* is the Euclidean distance between *a* and *b*, then *i* and *j* will make contact provided that *d* <*r*_*i*_(*a*) and *d* <*r*_*i*_(*b*). We assume that all individuals are aware of their own status (susceptible, infected, or recovered) and take this into account when deciding how to respond. Susceptible individuals reduce their contact neighbourhood because they know they are at risk of becoming infected, whereas infected and recovered individuals no longer have this risk and so do not reduce their contact neighbourhood.

### Economic benefit

To compare the effectiveness of different control strategies we measure the net economic benefit of applying the control. There are two aspects to be considered. Firstly, the control produces an economic gain if it causes a reduction in the overall impact of the epidemic. To measure this, we look at the number of recovered individuals at the end of the epidemic, *R*_*∞*_, and take the difference between the cases where control is used and where it is not used

(2) $$R_{\text{diff}}=R_{\infty }\;\text{(no control)}-R_{\infty }\;\text{(control)}\;.$$

The value of *R*_*∞*_*(no control)* is obtained by taking the mean of 20 simulation runs where control is not used. Similarly, *R*_*∞*_*(control)* is the mean of 20 runs with control.

Secondly, the control produces an economic loss by reducing the number of economically beneficial contacts taking place between pairs of individuals. To count the contacts taking place during an epidemic, we sum up the number of contacts that take place at each time step, over a fixed reference period. This reference period is chosen to be equal to 900 time steps which exceeds the duration of the longest epidemic in our sample (most epidemics were significantly shorter than 900 steps). We identify the economic benefits with the infection neighbourhood. This is equivalent to the assumptions that each economically significant contact is associated with an infection risk.

We assume that infected and recovered individuals are unaffected by the control, as they are no longer in danger of becoming infected, and that they therefore adopt the maximum contact radius, $r_{i}^{\left(0\right)}$. Susceptible individuals, however, may temporarily adopt a reduced contact radius, as explained above. This leads to a reduction in the total number of contacts when control is used, compared to the case when it is not used,

(3) $$C_{\text{diff}}=\text{contacts (no control)}-\text{contacts (control)}\;.$$

For any given $r_{i}^{\left(0\right)}$, the value of *contacts (no control)* is fixed: each individual will make contact with all of its neighbours within radius $r_{i}^{\left(0\right)}$, so a fixed number of contacts occurs at each time step, and *contacts (no control)* can be calculated by multiplying this number by the length of the reference period. The value of *contacts (control)* is obtained by taking the mean of 20 simulation runs with control.

The overall net benefit of using the control is then calculated by combining the gain from reducing *R*_*∞*_ with the loss from reducing the number of contacts. The parameter *c* represents the relative weight of a single contact compared to a single individual being saved from infection. So, for example, if we consider a single contact taking place to be equally valuable as a single case of illness that is prevented, then we would take *c*=1. If, however, a contact is considered to be more (or less) valuable than a saved infection, then *c* would be chosen to be correspondingly higher (or lower). The net benefit, *Δ*is calculated as

(4) $$\Delta =R_{\text{diff}}-c\times C_{\text{diff}}\;.$$

### Small-world model

In order to construct the small-world model [36,37], we start with the local-spread model as described above. A fixed number of local links (representing a proportion of all local links) is selected and those connections are ‘rewired’ to a random location outside the interaction neighbourhood determined by $r_{i}^{\left(0\right)}$, thus keeping the total number of links (local and long-range) constant. For simplicity we assume that the probability of passing an infection along any of the long-range links is the same as for local links.

As described above, in response to infection located in their awareness neighbourhood susceptible individuals reduce their number of local links. We assume that a similar behaviour governs small-world links. Thus, at each time step the number of active long-range links originating from a susceptible individual is proportional to (1−*Θ*^*α*^), where *Θ*represents the infection pressure within the awareness neighbourhood of this individual (as for local links). Infection can only pass along the long-range link if it is active and joins a susceptible and an infected individual. Finally, we assume that each active long-range link contributes to the calculation of the number of contacts in the same way as local links. Non-active long-range links do not contribute to the overall number of contacts.

### Simulations

**Figure 4:**
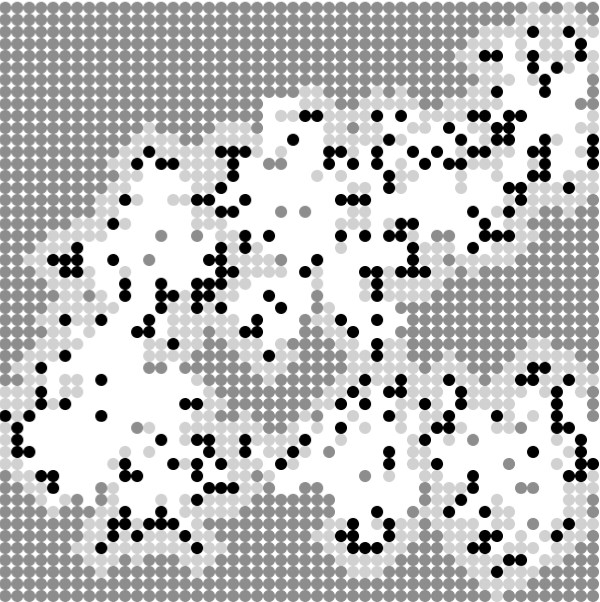
**Snapshot of a simulation run.** As the epidemic progresses, susceptibles (grey) close to infected (black) individuals become aware of their presence and reduce their social contacts. This results in a wall of cautious (light grey) susceptibles surrounding the infecteds, containing the epidemic and preventing it from reaching those further away. White areas indicate recovered individuals.

The model was implemented in NetLogo 4.0.4 [40], a simulation tool for agent-based stochastic simulation. Figure 4 shows a snapshot of a typical simulation run in NetLogo. Each run was replicated 20 times, so that in total over 2 million simulation runs were performed. Multiple, concurrent simulation runs were carried out on a grid of about 175 PCs with the help of the Condor distributed computing tool [41]. The statistical package R [42] was used to analyse the resulting data.

Simulation runs were performed for a spatial network consisting of a 50×50 square lattice without edge wrapping (total number of individuals, *N*=2500), with a fixed proportion of local links rewired to form a small-world model as described above. Table 1 lists the parameters and the values used in simulation runs. In order to obtain general results we cover a wide range of values for all parameters as shown in Table 1. The specific values shown in the figures have been chosen to indicate clearly the shape of our general results.

**Table 1:** Parameters of the model

****Parameter****	****Value****	****Interpretation****
*p*	0.01 − 1	probability of infection per contact (per time step)
*q*	0.01 − 1	probability of removal/recovery (per time step)
*r*_*i*_	1 − 5	contact radius (baseline)
*r*_*a*_	1 − 5, 10, *∞*	awareness radius
*α*	0.01 − 1.0	risk attitude
*c*	0.01, 0.1, 1, 10	Relative cost
*I*(0)	10 − 400	Initial number of foci (0.4% − 40%)

Without loss of generality, the time step is assumed to be one day.

## Results

**Figure 5:**
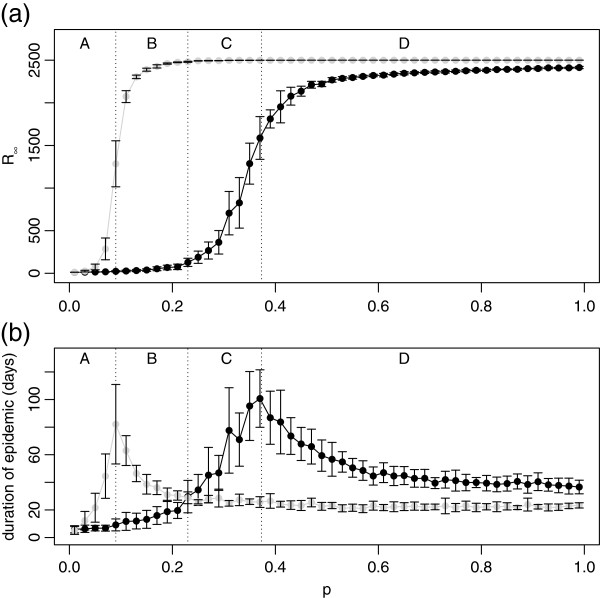
**Effect of control on the final size of the epidemic, ***R***_***∞***_(a) and epidemic duration,*T*(b).** Black lines indicate the result with control, and light grey indicates the result with no control. Each point is the mean of 20 simulation runs with error bars showing ±1 standard deviation. The parameter values are: *q* = 0.5, $r_{i}^{\left(0\right)}=2$, *r*_*a*_ = 2, *α* = 0.25.

We first describe results for the regular networks. In the absence of social distancing, the long-term behaviour of the spatial model depends on −, the probability that infection is passed to a susceptible individual from any of its neighbours within the infection neighbourhood, see Figure 5. For small values of *p*, the epidemics are short and limited (small epidemic duration, *T*, and small final epidemic size, *R*_*∞*_). When *p* reaches the threshold value (here *p*≃0.1), the epidemic rapidly increases and the duration time becomes very long. This slowing down is associated with a critical epidemic, lasting a very long time but creating relatively few infecteds at each time step. For large values of *p*≫0.1, the epidemic becomes very short but very rapid, with most individuals becoming infected (**I**) and eventually recovered (**R**). This picture is consistent with traditional models of disease spread on lattices and the corresponding percolation model [43].

### Effect of social distancing

Social distancing (characterised here by a relatively risk-averse attitude, *α*=0.25, see Figure 3) shifts the critical infectiousness *p* towards higher values but also generally increases the duration of the epidemic, Figure 5. For a given *p*, social distancing always decreases the final size of the epidemic (Figure 5a), but the effect on the duration depends on the value of *p* (Figure 5b). The different outcomes are indicated roughly by the four regions marked on the graphs, labelled A, B, C, and D, and with the approximate boundaries between them shown as vertical dotted lines in Figure 5 and in other figures below. For small values of *p* the disease is non-invasive in both cases, while both the final size and the duration of the epidemic are lower in presence of social distancing than without it (region A). In regions B and C, the epidemic without social distancing is invasive, but the behavioural changes render it non-invasive. However, in region B the duration of the epidemic is shortened by the distancing, whereas in region C it is longer. Finally, in region D the disease is invasive regardless of the social distancing, with slightly lower final size, but significantly longer duration. The approximate boundaries between regions are given by the transition between invasive and non-invasive disease for the case without control (boundary between A and B) and with control (boundary between C and D), respectively. In addition, the boundary between B and C is placed at the value of *p* where controlled and uncontrolled epidemics last approximately the same time, see Figure 5.

**Figure 6:**
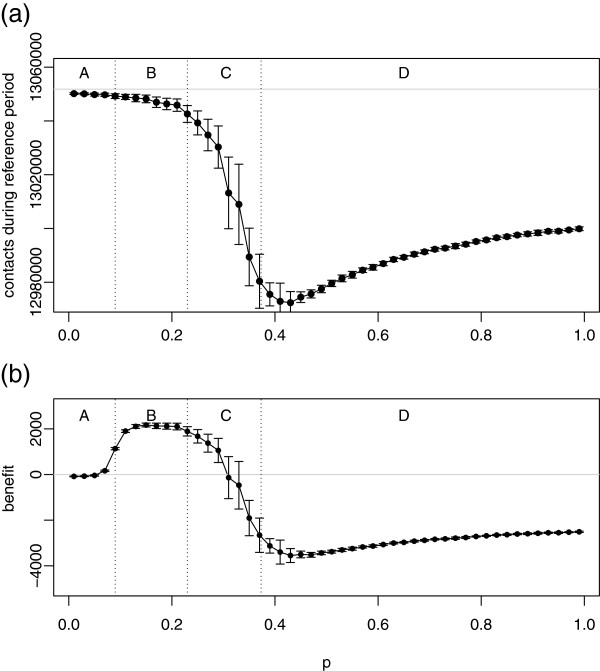
**Number of contacts (a) and net economic benefit (b) as functions of*p*.** Black lines indicate the result with control, and light grey indicates the result with no control. Each point is the mean of 20 simulation runs with error bars showing ±1 standard deviation. The parameter values are: *q* = 0.5, $r_{i}^{\left(0\right)}=2$, *r*_*a*_ = 2, *α* = 0.25, and *c* = 0.05.

The overall number of contacts taking place during an epidemic is affected by two factors: epidemic size and duration. Firstly, the size of the epidemic determines how many individuals are still susceptible, and therefore exercising the control (infected and recovered individuals do not change their behaviour in our model). A larger epidemic also creates a higher infection pressure, leading to increased reaction. Secondly, the longer the duration of the epidemic the more pronounced the cumulative effect of the reduction of contacts. Both factors combine to reduce the overall number of contacts differentially over *p*, see Figure 6a. The effect is relatively minor in regions A and B, but becomes very strong in region C, where the long duration of the epidemic combines with relatively large number of infections. Finally, in region D, although the epidemic leads to many cases, it lasts for a relatively short period of time. This results in a smaller drop in contact numbers.

The economic impact of the reduction in the number of contacts can be offset by a reduction in disease cases caused by the social distancing. The two factors are weighed by *c*, the relative cost of social interactions versus cost of infection. A typical dependence of the net economic benefit on *p* for medium values of *c* is shown in Figure 6b; we will explore the dependence on *c* later in the paper.

Overall, we see that the effect of the control may be neutral, beneficial, or detrimental, with the outcome depending on the infectiousness of the disease, *p*. The effect is different in different regions A-D. In region A the control makes little difference as the disease is not invasive even in the absence of social distancing. *R*_*∞*_ is small and the epidemic is of short duration. Using the control causes a very slight reduction in the number of contacts, and a correspondingly very small reduction in the net benefit.

As *p* increases past the uncontrolled epidemic threshold value, indicated by the boundary between regions A and B in the graphs, the social distancing leads to a positive net benefit. The control reduces *R*_*∞*_almost to zero, and greatly shortens the duration of the epidemic. Although the number of contacts is also somewhat reduced, this is more than compensated for by the large reduction in *R*_*∞*_, so that the net benefit is strongly positive.

As *p* increases further, region C, the control becomes ineffective, *R*_*∞*_ rises towards the levels seen without control, and the duration of the epidemic increases beyond no-control levels. The picture is of a control that is too weak to suppress the epidemic and is merely slowing down the speed of its spread without reducing its final impact. Figure 6a shows a further downside: by prolonging the epidemic, the control prolongs the period during which social distancing is practised, thus greatly reducing the number of contacts. This in turn results in an overall negative benefit. Eventually, as *p* enters region D, we reach the worst case scenario for the control, where using the control gives a much worse result than doing nothing. As *p* increases further beyond this point, we return to a situation where, as in region A, use of the control makes little difference to the severity of the epidemic or its duration. However, unlike region A, in this case the epidemic is invasive despite the control. The number of contacts and the overall benefit both improve slightly from the worst case scenario, though remaining low.

An interesting observation from these graphs is the close juxtaposition of the best case scenario with the worst case, as shown by the steep transition from positive to negative benefit taking place within region C. This implies that if social distancing is to be used to control disease, it is very important to get the parameters right. If the infectiousness of the disease is underestimated even slightly, the actual outcome of using the control can be substantially worse than anticipated. We shall see later on, when we consider different attitudes to risk and different values of the awareness radius, that the importance of getting the parameters exactly right is a recurring theme in this work.

### Effect of varying c

**Figure 7:**
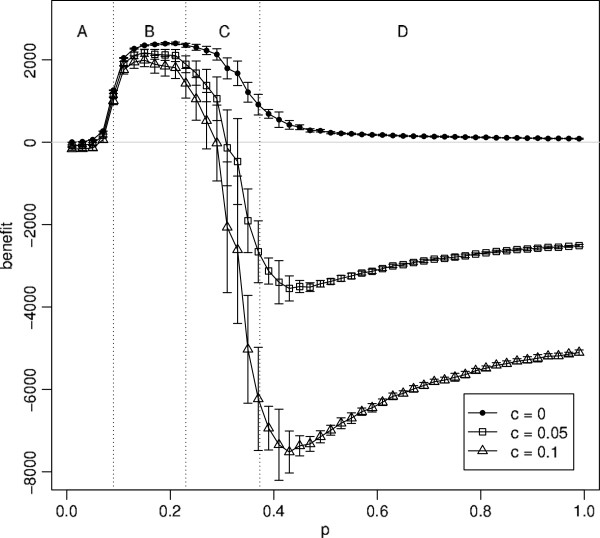
**Net economic benefit as a function of ***p, for ***c*** = 0***, ***c = 0.05 ***and ***c = 0.1***.** Each point is the mean of 20 simulation runs with error bars showing ±1 standard deviation. The parameter values are: *q* = 0.5, $r_{i}^{\left(0\right)}=2$, *r*_*a*_ = 2, and *α* = 0.25.

The parameter *c* represents the relative weight of a single contact between a pair of individuals, compared to a single case of infection. Changing *c* causes a vertical shift in the benefit, that is most significant in regions C and D. However it does not change the overall shape of the graph, Figure 7. If *c*=0 then there is no cost attached to reducing contacts, so using the control always results in a non-negative benefit (top line). As *c* is increased, reducing contacts has a greater impact, causing a strong downward shift in regions C and D of the graph where large numbers of contacts are lost. For the remaining graphs, *c*=0.05 is chosen because it best illustrates the generic behaviour of the net economic benefit.

### Importance of risk attitude

**Figure 8:**
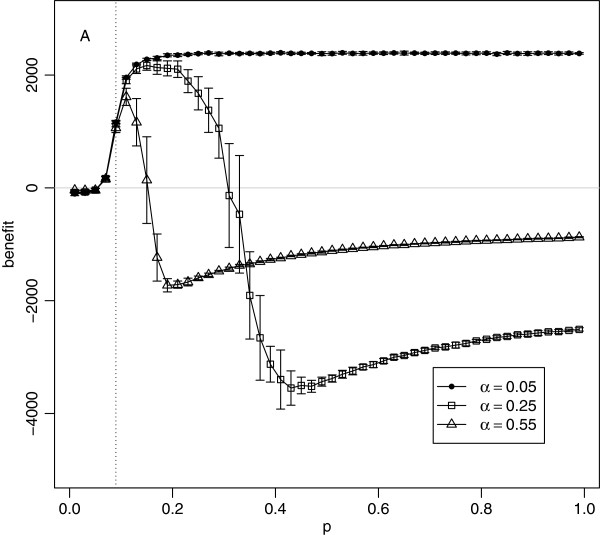
**Net economic benefit as a function of ***p, for ***α*** = 0.05***, ***α = 0.25 ***and ***α = 0.55***.** Each point is the mean of 20 simulation runs with error bars showing ±1 standard deviation. Parameters: *q* = 0.5, *c* = 0.05, $r_{i}^{\left(0\right)}=2$ and *r*_*a*_ = 2. Only region A is marked as the locations of regions B, C, and D vary with *α*.

The parameter *α*represents the attitude to risk that determines how social distancing is applied. A very low value of *α* means highly risk-averse behaviour, causing a strong control response in which susceptible individuals effectively close down all social contacts if there is any hint of disease within their awareness radius, Figure 3. Higher values of *α* represent more relaxed (less risk-averse) attitudes and will cause a weaker response, particularly at low infection pressure, *Θ*. When all individuals are highly risk averse (*α*= 0.05), social distancing leads to a positive benefit for even the most highly infectious diseases, see Figure 8. The only exception is region A where disease does not spread even in absence of social distancing, but changes in behaviour around the initial foci lead to slightly negative benefit. However, if the risk attitude is made more relaxed (*α* = 0.25 and *α*=0.55 in Figure 8), the control becomes less effective for more infectious diseases, resulting in a worse outcome than doing nothing.

Another way of thinking about these results is in terms of the four regions, A, B, C, and D, identified earlier. With a highly risk-averse value of *α*, regions C and D vanish, and region B (corresponding to the diseases for which the distancing is beneficial) extends from the invasion threshold value of *p* all the way to *p*=1. Relaxing (increasing) *α* causes region B to narrow and regions C and D to emerge. As *α*is increased further, the boundary between regions B and C shifts to the left, meaning that the control is beneficial for a narrower range of diseases.

**Figure 9:**
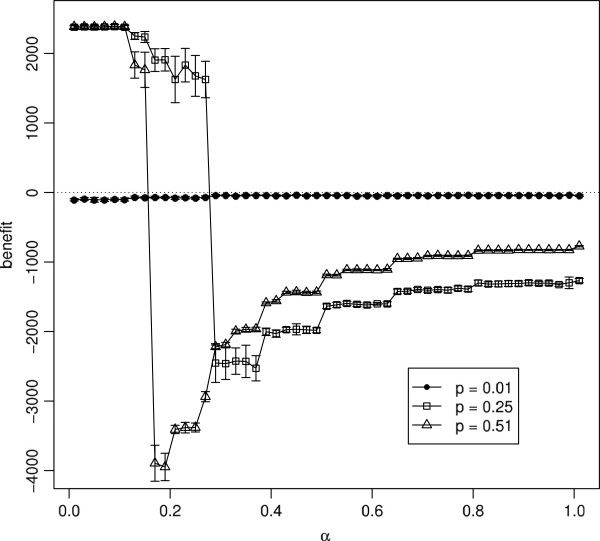
**Net economic benefit as a function of ***α, for ***p*** = 0.01***, ***p = 0.25 ***and ***p = 0.51***.** Each point is the mean of 20 simulation runs with error bars showing ±1 standard deviation. Parameters: *q* = 0.5, *c* = 0.05, $r_{i}^{\left(0\right)}=2$ and *r*_*a*_ = 2.

In Figure 9, we take a different view of this data, choosing three different diseases (represented by different values of *p*) and analysing how the benefit changes as *α* is varied. For a non-invasive disease (*p*=0.01), using the control has little effect and varying *α* makes little difference, although a high value of *α*represents the optimal choice corresponding to a maximum value of the benefit. For a moderately infectious disease (represented by *p*=0.25), there is a positive benefit for lower (more risk averse) values of *α*, up until a sharp threshold value, after which the outcome is negative. As *α* is increased further, the outcome improves, though remaining negative. These graphs imply that it is very important to exercise sufficient caution in using social distancing. If the control is just slightly too relaxed and falls on the wrong side of the threshold, the result will be worse than doing nothing. For a more infectious disease (*p*=0.51), control is only beneficial if *α*is extremely risk-averse.

### Small-world model

**Figure 10:**
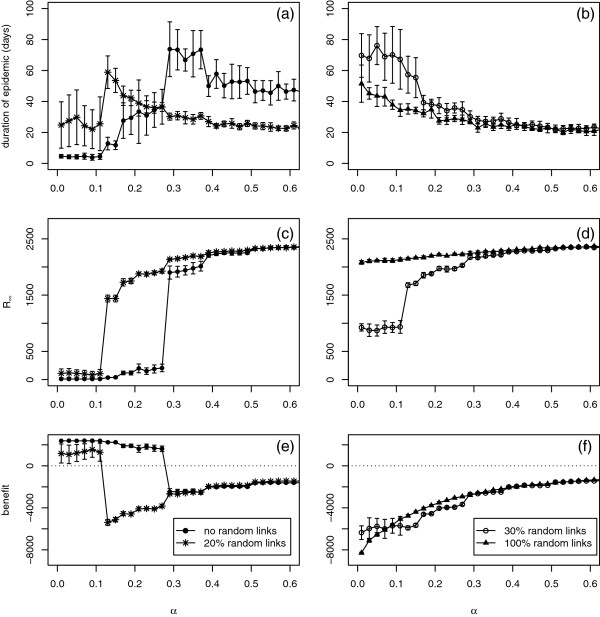
**Duration (a) and (b), the final size of the epidemic (c) and (d) and the net benefit (e) and (f) as a function of *α*for different numbers of long-range random links.** Each point is the mean of 20 simulation runs with error bars showing ±1 standard deviation. Parameters: *p* = 0.25, *q* = 0.5, *c* = 0.05, $r_{i}^{\left(0\right)}=2$ and *r*_*a*_ = 2. 20% corresponds to 3,000 links, 30% to 4,500 and 100% represents a fully random network.

For the case described above when only short-range links are present in the network, the disease spreads locally and can therefore be controlled efficiently by individuals reducing their social activities. Addition of randomness increases the propensity of the disease agent to spread by allowing it to create new foci in regions where there is a high density of susceptible individuals. As a result, the switch from the invasive to the non-invasive disease occurs for smaller values of *p*[13,36]. The long-range links can also make social distancing less effective, as individuals are responding to infection within their local neighbourhood and the disease can pass along the long-range links from outside this neighbourhood. However, even when 20% (3,000) of links are long-range, this does not render the control completely ineffective, Figure 10 (left column), although it requires a more cautious attitude (smaller *α*) for it to work. For networks dominated by long-range spread (as exemplified here by a small-world network with 30% long-range links and by a completely random network), the social distancing breaks down and the optimal solution is to avoid any reaction to the disease, Figure 10 (right column).

The process by which inclusion of long-range links breaks down the usefulness of the social distancing is, however, not trivial. Two contrasting cases need to be distinguished, corresponding to a very relaxed risk attitude (*α*∼1) and to a very strict response (*α*∼0), see Figure 10. Firstly, if individuals do not respond to the local infection load (large *α*), the epidemics are usually very quick (rightmost parts of Figure 10a,b) and infect most of the individuals in the population (Figure 10c,d). However, the number of contacts lost to social distancing is also small, resulting in the net benefit close to 0 (rightmost parts of Figure 10e,f).

As the individuals become more cautious in their response to the infection load, the duration of the epidemics goes up (Figure 10a) and the number of cases goes down (Figure 10c). In the second case, where the values of *α* are small, the behaviour depends crucially on the number of long-range links. For small number of such contacts, the epidemics are short (leftmost parts of Figure 10a) and stop after infecting a small number of individuals (leftmost parts of Figure 10c). As the number of long-range links increases to 30%, the duration of the epidemic increases (Figure 10b) and the number of cases goes up (Figure 10d) but remains short of infecting the whole population. In this case, epidemics progress slowly, infecting only few individuals at a time, but resulting in a massive reduction in the number of contacts over the whole period of their duration. Further addition of non-local links results in a faster epidemic affecting a large number of individuals (Figure 10b,d). For the particular choice of *c* in Figure 10 this results in a very similar value of the net benefit for 30% and 100% non-local links, although the balance will change when other values of *c* are selected (cf. Figure 7).

In this model we assume that the control is applied to both local and long-range links in the same way. If long-range links are excluded from the control, it becomes even less effective, but the results are qualitatively similar to those described above.

### Importance of matching spatial scales: regular networks

**Figure 11:**
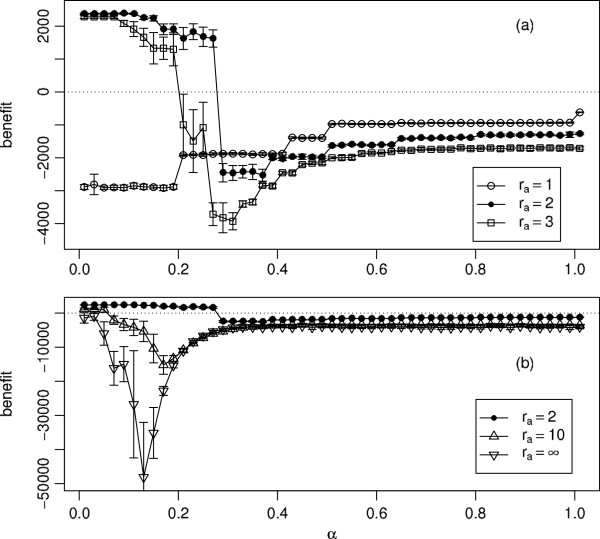
**Net economic benefit as a function of ***α***, for different values of ***r***_***a***_.** Each point is the mean of 20 simulation runs with error bars showing ±1 standard deviation. Parameters: *p* = 0.25, *q* = 0.5, *c* = 0.05, $r_{i}^{\left(0\right)}=2$ and *r*_*a*_ takes the values 1, 2 (= $r_{i}^{\left(0\right)}$), and 3 in (**a**) and 2 (= $r_{i}^{\left(0\right)}$), 10 and *∞ *in (**b**). Note the change of vertical scale between (**a**) and (**b**). *r*_*a*_ = *∞ *is modelled by making the awareness neighbourhood sufficiently large to include the whole population.

The model introduces two spatial scales, the infection (and contact) neighbourhood and the awareness neighbourhood. The infection neighbourhood is associated with disease transmission and with economic benefits accruing from social contacts, whereas the role of the awareness neighbourhood is to provide an estimate of risks associated with infection. So far in the case of regular networks we have only considered cases in which the radius of the awareness neighbourhood *r*_*a*_ is equal to that of the maximum contact radius $r_{i}^{\left(0\right)}$. This corresponds to a situation when individuals base their decisions on the same social neighbourhood as the infection risk comes from. However, two other cases need to be considered. The individuals might be in contact potentially leading to infection with individuals whose status might be unknown to them. In our approach we simulate this situation by considering an awareness neighbourhood which is smaller than the infection neighbourhood. This results in underestimation of risks and leads to sub-optimal control of epidemic spread, as illustrated by $r_{a}=1<2=r_{i}^{\left(0\right)}$ in Figure 11a. The benefit is in this case a monotonically increasing function of *α*, leading to a maximum corresponding to a relaxed attitude (large *α*).

This behaviour is very different to the cases when $r_{a}\geq r_{i}^{\left(0\right)}$, i.e. when the individual bases the decisions on a sample equal or larger than the infection neighbourhood. The benefit is a non-monotonic function of *α* with a global positive maximum at low values of *α*, a rapid drop to highly negative values for intermediate *α*and improving for large *α*, Figure 11. However, extension of the awareness neighbourhood beyond $r_{i}^{\left(0\right)}$ reduces the range of *α*for which the benefit is positive and significantly lowers the minimum value of the benefit (Figure 11). In particular, if the awareness neighbourhood includes the whole population, *r*_*a*_=*∞*, the benefit is negative for all values of *α*considered here. Two mechanisms may contribute to produce this lack of efficiency. Firstly, it might be that individuals reduce contacts unnecessarily in response to awareness of disease cases that are too far away to pose a threat. Alternatively, it might be that individuals fail to protect themselves because the over-large awareness radius causes them to underestimate the level of infection amongst their local contacts. Examination of the simulation data suggests that a combination of these two mechanisms is responsible for this result, because making *r*_*a*_ larger than $r_{i}^{\left(0\right)}$ has the dual effect of causing an increase in *R*(*∞*) and a reduction in the number of contacts. Thus, the exact match of the awareness and infection neighbourhood appears to lead to an optimal case with the widest range of *α*for which benefit is positive and the shallowest drop in the benefit for intermediate values of *α*.

### Importance of matching spatial scales: small-world networks

**Figure 12:**
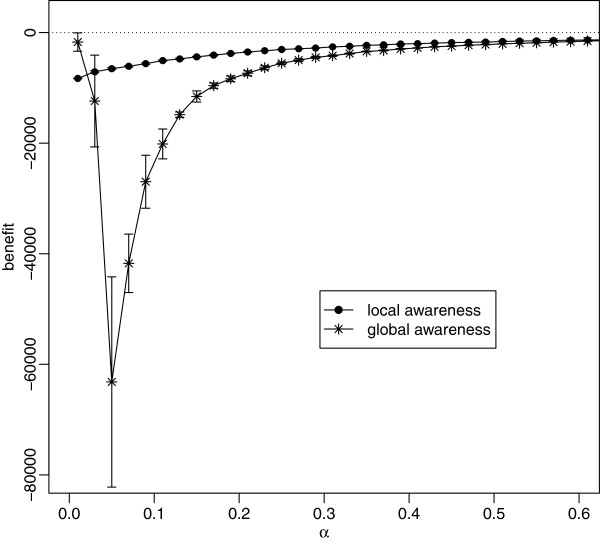
**Net economic benefit as a function of *α***, **for *r***_***a***_** = 2 (local awareness) and *r***_***a***_ = *∞ ***(global awareness) for fully random network.** Each point is the mean of 20 simulation runs with error bars showing ±1 standard deviation. Parameters: *p* = 0.25, *q* = 0.5, *c* = 0.05, $r_{i}^{\left(0\right)}=2$.

Long-range links provide an alternative way in which disease can enter the local neighbourhood of the susceptible individuals. The question then arises whether global awareness of infection cases is able to restore the efficiency of social distancing when the network is dominated by random links? Figure 12 which shows the results for the random network suggests that this is not the case; the results for other numbers of non-local links are very similar. The loss of contacts resulting from over-reacting to infection far dominates the net benefit. However, for random networks with a very cautious response (very small *α*) global awareness is more efficient than local awareness, see Figure 12.

## Conclusions

Within the assumptions of our model, we have seen that social distancing is most cost-effective as an epidemic control strategy if it is applied with a high degree of caution (i.e., a low risk attitude), and that the more infectious the disease, the greater the caution that is required. If a disease is very highly infectious, social distancing may have no effect, or may require an unfeasibly high degree of caution in order to be effective. In these cases, doing nothing will be a more cost-effective strategy than using social distancing, because the worst-case outcome arises if the control is applied, but the level of caution used is too weak. The steep threshold separating the best-case outcome from the worst-case in Figure 6b indicates that it is very important that the level of infectiousness of the disease is not underestimated. The similar, steep threshold in Figure 9 highlights the danger of having an overly relaxed attitude to risk when using the control. Hence the slogan in the title of this paper: if social distancing is being considered, do it well, or not at all.

We have also seen the importance of having a good match between the spatial area from which an individual draws information which will affect his/her behaviour, and the spatial area containing those other individuals who actually pose an infection risk. The control works best when these two areas are the same. If the individual has too little knowledge and can be infected by others who are outside of the awareness radius, the control does not work. If, on the other hand, the individual responds to infection cases which do not pose a realistic risk of infection (as might happen if, say, individuals in the UK change their behaviour in response to mass media reports of faraway epidemics in distant countries) the control is also inefficient.

Additional simulation runs were performed to check the robustness of our results. We looked at the results of varying the number of initial foci from 0.4% to 40% of the population (10-1000 infected individuals in population of 2500). As the number of foci is increased, the epidemic reaches more of the population and its duration is shortened. The control becomes less effective, with fewer contacts taking place during the reference period and a reduction in the economic benefit from using the control. However, the overall shape of the results does not change. We also checked potential effects of different size and boundary conditions by performing exploratory runs with different assumptions. Edge wrapping has the effect of slightly increasing *R*_*∞*_ and reducing the duration of the epidemic, but the overall shape of the graphs remains the same. Varying the size of the system (ranging from 400-10000 individuals) has no effect on the results. We also varied the recovery rate, *q*, in the range from 0.1 to 0.9; with appropriate scaling of *p* the results are consistent with those shown above. Finally, the simulations for small-world networks (up to 30% of long-range links) and a random network suggest that our results are fairly robust with respect to making the network structure more realistic.

Care must be taken in interpreting our results and attempting to relate them to epidemics in the real world. The model relies on a number of assumptions which must be taken into account. First, the disease model is a simple SIR model. Further work needs to be done to investigate whether the results hold for other systems, such as SIS or SEIR models and for real-world social networks. Another major assumption concerns the way we deal with recovered and infected individuals: we have supposed that these individuals maintain full contact with others, and that only susceptible individuals change their behaviour. If we changed these assumptions, for example, by assuming that recovered individuals die and cannot therefore engage in economic contact, or by assuming that infected individuals are weakened and have fewer contacts than healthy individuals, then our results might differ. This, again, is a subject for future investigation. Our present conclusions can only be applied to mild diseases which do not greatly affect the behaviour of infected individuals, and do not typically result in death.

The model can be extended in a number of ways. Currently we assume that the population is constant and that recovered individuals cease to be infectious and cannot be infected again. These assumptions can be relaxed to include full recovery, births, deaths and other demographic processes as well as asymptomatic stages of the disease or indeed asymptomatic carriers. In our model we do not distinguish between different activities and hence combine day/night cycles into one time step. Similarly, we do not include variations in contacts between weekdays and weekends or schools/holidays. Finally, with increased availability of data it is possible to analyse existing social networks in unprecedented detail. Each of these extensions is possible in agent-based models like ours, but exceeds the scope of the paper.

In our approach we have made a simple assumption about the monetary value of each contact (represented by *c*) and we assumed that all contacts (whether for monetary or non-monetary reasons) have the same value. This is clearly only a first approximation and an obvious extension of our model (beyond the scope of the current paper) is to consider different monetary value for different contacts. More work is also needed on understanding how individuals value different aspects of their social and economic activities, in the light of a possibility of losing them due to disease control. For simplicity we have also assumed that the only difference between the long-range links and the local links is in the topology of interactions; otherwise the long range links are treated the same as short-range links, in terms of reduction of contacts in response to infection and their contribution to the individual well-being. This is again a simplifying assumption as the long-range links may be more likely to be broken in the presence of infection. Alternatively, individuals trying to get away from the epidemic foci might increase the propensity of long-range links, as documented in e.g. [44].

When long-range links are included in the network, there are three options for the control. Authorities can either block the travel or strongly advise against it and individuals might respond to the epidemic threat by completely refraining from such activities, in which case the model becomes purely local. At the other extreme, individuals might not have control over occasional long-range contacts and the changes in the behaviour will be purely local. Finally, a mixture of responses is possible, with different behaviour associated with different kinds of links. In our paper we considered all three cases, although for the last one we assumed that local and long-range links are treated in the same way.

Other areas for future work could involve extending the economic and psychological aspects of the model to capture further aspects of human behaviour [34,45]. Economic models, for example, often include the phenomenon of time discounting, whereby losses or gains anticipated to take place in the long term are considered less important than those occurring in the short term. This phenomenon could be included in models of lengthy epidemics where discounting may play a role in the decision-making processes of both individuals and social planners. Another aspect we have not considered is the role of memory and habitual behaviour; in our model, individuals respond instantaneously to the current conditions and do not remember what has happened in the past. Other researchers [46] have incorporated memory into models of behavioural response to epidemics, and this is something that could usefully be added to our model.

The results of this paper raise the question of how individuals respond to the threat of disease in the real world. There is surprisingly limited quantitative data available about changes in social interactions during historical epidemics. Surveys and questionnaires can provide static snapshots of behaviour but only limited information about changes over time and in response to infection pressure. For obvious reasons, it is not possible to run experiments involving induced epidemics in the real world. A promising alternative which we are currently pursuing is to use virtual experiments based on agent-based simulations of epidemics in which human beings participate and control the responses of the agents [47].

## Competing interests

The authors declare that they have no competing interests.

## Authors’ contributions

SM and AK contributed equally to the study design and modelling as well as preparation of the final manuscript.

## Pre-publication history

The pre-publication history for this paper can be accessed here:

http://www.biomedcentral.com/1471-2458/12/679/prepub
